# Short-term impact of the COVID-19 confinement measures on health behaviours and weight gain among adults in Belgium

**DOI:** 10.1186/s13690-021-00542-2

**Published:** 2021-02-22

**Authors:** Sabine Drieskens, Nicolas Berger, Stefanie Vandevijvere, Lydia Gisle, Elise Braekman, Rana Charafeddine, Karin De Ridder, Stefaan Demarest

**Affiliations:** 1 Scientific Direction Epidemiology and public health, Sciensano, J. Wytsmanstreet 14, 1050 Brussels, Belgium; 2 Population Health Innovation Lab, Department of Public Health, Environments & Society, London School of Hygiene & Tropical Medicine, London, WC1H 9SH UK; 3 Faculty of Public Health, University of Louvain, Brussels, Woluwe Belgium

**Keywords:** Confinement, COVID-19, Weight gain, Change in health behaviours

## Abstract

**Background:**

In Belgium, confinement measures were introduced on the 13th of March 2020 to curb the spread of the coronavirus disease (COVID-19). These measures may affect health behaviours of the population such as eating habits, physical activity and alcohol consumption, which in turn can lead to weight gain resulting in overweight and obesity, increasing the risk of several chronic diseases, but also of severe COVID-19. The purpose of this study is to assess the impact of confinement measures on health behaviours and their associations with weight gain.

**Methods:**

Data were derived from the second national COVID-19 health survey. Data were collected between the 16th and the 23rd of April 2020. The recruitment of participants was based on snowball sampling via Sciensano’s website, invitations via e-mail and social media. The study sample includes participants aged 18 years and over with no missing data on the variables of interest (*n* = 28,029). The association between self-reported weight gain and health behaviour changes, adjusted for gender, age group and household composition was assessed through OR’s (95% CI) calculated with logistic regression models, using post-stratification weights.

**Results:**

Overall, 28.6% reported weight gain after 6 weeks of confinement. Higher odds of weight gain were observed among participants who increased or decreased their consumption of sugar-sweetened beverages (OR = 1.39 (1.15–1.68) and 1.29 (1.04–1.60), respectively), among those who increased their consumption of sweet or salty snacks (OR = 3.65 (3.27–4.07)), among those who became less physically active (OR = 1.91 (1.71–2.13)), and among those who increased their alcohol consumption (OR = 1.86 (1.66–2.08)).

**Conclusions:**

The most important correlates of weight gain during confinement were an increased consumption of sweet or salty snacks and being less physically active. These findings confirm the impact of diet and exercise on short term weight gain and plead to take more action, in supporting people to achieve healthier behaviours in order to tackle overweight and obesity, especially during the COVID-19 pandemic.

## Background

In Belgium, several confinement measures were introduced by the National Security Council on the 13th of March 2020 with the aim of curbing the spread of the coronavirus disease (COVID-19) [1]. The confinement measures included among others the closure of hotels, bars and restaurants as well as schools/universities, non-essential industries and the restriction of cultural, recreational and sports activities. Teleworking became the norm whenever possible, non-essential movements were forbidden and the borders were closed. Parks and other green spaces generally remained open but were subject to strict physical distancing and any form of group gatherings was forbidden. A gradual loosening of the confinement measures started on the 4th of May 2020.

Confinement measures may impact health behaviours, such as eating habits and physical activity, both in a positive and in a negative way [2, 3]. If sustained over a long period of time, changes towards unhealthy behaviours may then affect the health status of the population. Specific health behaviours such as overeating, unhealthy diet and reduced physical activity may contribute to weight gain [4]. Besides, there is also evidence that alcohol consumption is associated with an increase in the Body mass index (BMI) [5]. Weight gain may lead to overweight and obesity, which may enhance the risk of cardiovascular diseases, type 2 diabetes and some cancers, and consequently premature mortality, which makes it a major public health problem [6]. Moreover, recent studies have shown that obesity increases the risk of severe COVID-19 (more respiratory complications) and consequently a longer stay in the hospital [7–9].

One of the key risk factors for weight gain that may be affected by confinement measures is unhealthy eating habits. Snacking is the intake of specific foods, often nutrient-poor, energy-dense, between traditional meals. Epidemiological studies have found a positive association between snacking and weight gain among adults [10, 11]. Often, bad nutritional habits are related to a higher consumption of sugar-sweetened beverages, also a risk factor for obesity [12].

Being more often at home during confinement, may give easier access to snacks – often also a cheaper alternative to healthier options – and sugar-sweetened beverages, and extra occasions to consume them.

Furthermore, a high consumption of food prepared out-of-home has also been related to weight gain [13, 14]. The confinement may have affected out-of-home food consumption (i.e. closure of restaurants, except home delivery options) and so possibly weight status. However, some countries have found that certain subpopulations may eat healthier during confinement [2, 15, 16]. For example, a Dutch study has shown that younger adults tended to spend more time cooking healthier food, to eat more fruits and vegetables, and to have less unhealthy temptations which usually take place during social gatherings, at work, or during commuting [17].

Another important health behaviour that may be impacted by the confinement is physical activity. Potential reasons for a decrease in physical activity include the fact that people are recommended to stay at home which reduces their movements. Besides, the closure of indoor sport facilities, as well as the combination of work and homeschooling may be additional factors for reducing physical activity [18]. The association between physical inactivity and obesity is well documented [19–21]. Conversely, some people may have had more time to be physically active during the confinement period.

The last risk factor for weight gain that may be impacted due to the COVID-19 confinement is the consumption of alcohol. Studies have generally shown that light to moderate alcohol consumption is not associated with obesity, but heavy drinking and binge drinking are [22]. This can be explained by the high sugar level in some alcoholic drinks and the fact that alcohol stimulates the craving for and intake of unhealthy foods [22–24]. It is expected that an increase in psychosocial distress during confinement might have increased alcohol consumption for some, while deteriorating financial situation and reduced availability of onsite alcohol areas, such as bars, might have reduced consumption for others [25].

The purpose of this study is to assess the short-term impact of the COVID-19 confinement measures on health behaviours, such as eating habits, physical activity and alcohol consumption, and on the change in body weight among adults in Belgium. Further, the associations of these health behaviours with weight gain during confinement were determined.

## Methods

### Survey methodology

To evaluate the impact of the confinement measures on the mental health, health behaviours and weight status of the population, Sciensano, the Belgian institute of public health, organised a series of online health surveys. The first COVID-19 health survey was launched 3 weeks after the start of the confinement period (the 2nd of April), the second survey took place 2 weeks later (the 16th of April), the third one started on the 28th of May, the fourth on the 24th of September and finally the fifth on the 3rd of December 2020. All five surveys were developed using LimeSurvey version 3 and were available online for 1 week. The launch of the surveys and the call for participation were announced on the website of Sciensano and of other organisations (health insurance organisations, community centres …), and at the COVID-19 press conference, through the press and on social media. Recruitment was based on snowball sampling [26]: participants were asked to share the link of the survey with their family, friends and acquaintances. Participants who had indicated in a given survey that they would like to take part in the next one received an invitation through the e-mail address they provided. This cross-sectional survey was approved by the ethical committee of the University of Ghent (BC-07544). Before participants could start with the survey, they had to indicate that they lived in Belgium, were at least 18 years old and gave their consent to six terms and conditions including voluntary participation, confidentiality of the data and the right to withdraw at any time. This was done in order to be in line with the General Data Protection Regulation (GDPR) and the Declaration of Helsinki [18].

### Study population

The data for the purpose of this study were derived from the second COVID-19 health survey that included specific questions on health-related behaviours. After exclusion of participants with missing data on the sociodemographic covariates and health behaviour indicators, the final study sample contained 28,029 individuals aged 18 years and older. Since the study sample was biased at the level of region (underrepresentation of the Flemish Region and overrepresentation of the Walloon Region), gender (overrepresentation of women), age group (underrepresentation of the youngest (18-24 years) and oldest (65+ years)) and educational attainment (underrepresentation of the low educated), post-stratification weights taking these elements into consideration were applied. The post-stratification technique uses information on the composition of the population from another data source to rebalance the sample. Population in terms of gender, age, province of residence and educational attainment benchmark were obtained with reference to the “Labour Force Survey” carried out by Statbel in 2018 [18].

### .Variables

Table 1 gives an overview of the health-related survey questions, their answer categories and the derived indicators (description and construction of the categories).

**Table 1:** Overview of the self-reported health questions, their answer categories used in the second COVID-19 Health Survey and the related indicators, Belgium 2020

Questions	Answer categories	Indicators: description	Indicators: categories
How tall are you without clothes and shoes?	Length in centimeters	Weight status	1. Underweight (BMI < 18.50) 2. Normal weight (BMI = 18.50–24.99) 3. Overweight (BMI = 25.00–29.99) 4. Obesity (BMI ≥ 30.00)
How much do you weigh without clothes and shoes?	Body weight in kilogram		
Since 13 March 2020, has your body weight changed?	1. Yes, lost weight 2. Yes, gained weight 3. No, my body weight remained stable 4. Don’t know	Weight gain	1. Yes (category 2) 2. No (categories 1 and 3)
Since 13 March 2020, has the consumption of the following foods increased, remained unchanged or decreased? - sugared-sweetened beverages, i.e. lemonade, cola or ice tea (no ‘light’) - Sweet or salty snacks such as candy, chocolate, cake, biscuits, ice cream, chips,... - Food prepared out-of-home such as fries, sandwiches, takeaway, home delivery via apps, caterer,...)	1. Increased 2. Remained unchanged 3. Decreased	Change in the consumption of sugared-sweetened beverages Change in the consumption of sweet or salty snacks Change in the consumption of food prepared out-of-home	1. Increased 2. Unchanged 3. Decreased
Since 13 March 2020, have you changed your physical activity habits (walking, cycling, sports...)?	1. I’ve never done physical activity and now neither 2. I’ve never done any physical activity, but I’ve started now 3. I’m doing more physical activity now 4. I do as much physical activity 5. I’m doing less physical activity now	Change in physical activity	1. Increased (categories 2 and 3) 2. Unchanged (categories 1 and 4) 3. Decreased (category 5)
Since 13 March 2020, have you modified your usual consumption of alcohol?	1. I don’t use 2. I started using (again) 3. More than usual 4. Less than usual 5. Same as usual 6. I stopped using since then	Change in alcohol consumption	1. Increased (categories 2 and 3) 2. Unchanged (categories 1 and 5) 3. Decreased (categories 4 and 6)

### Health indicators

The outcome measure was self-reported ‘weight gain’ over 6 weeks during confinement (Table 1). The five health behaviour indicators were ‘change in the consumption of sugar-sweetened beverages’, ‘sweet or salty snacks’, ‘food prepared out-of-home’, ‘change in physical activity’ and ‘change in the consumption of alcohol’. Response categories were classified as ‘increased’, ‘unchanged’ and ‘decreased’. The BMI (kg/m^2^) was calculated based on self-reported height and weight. The weight status was classified as underweight, normal weight, overweight and obesity using WHO BMI cut-offs [27].

### Sociodemographic and health covariates

Amongst the sociodemographic variables measures in the survey, we identified the variables which could have a possible impact on weight gain. Retained sociodemographic covariates were: gender (men and women), age group (18–24, 25–34, 35–44, 45–54, 55–64 and 65+ years), education attainment (secondary school diploma or less versus higher education), household composition (living alone; couple without child(ren); couple with child(ren); living alone with child(ren); living with parents, family, friends; other) and employment status (no paid job,^1^ paid job conducted at the normal work place, paid job via telework, paid job but temporarily interrupted and paid job in another situation). Since also the health status can act as a confounder, a quality of life indicator (EQ-5D), was defined as covariate. This indicator combines 5 dimensions (problems in mobility, self-care, performance of the usual activities, pain/discomfort and anxiety/depression) of a standardized instrument (scale) developed by the European EuroQol group and makes a distinction between people who have no health problem versus people who have at least one health problem.

### Data analysis

In first instance, it was tested if the covariates were individually associated with weight gain (*P* < 0.05). This was the case for all covariates, except for educational attainment (low versus high), and therefore the latter was no longer taken into account. The weighted distribution (in percentage) of the covariates and the health indicators among the study population was determined in a weighted frequency table. Next, the percentage of population reporting weight change over 6 weeks during confinement was reported by weight status. The association between self-reported weight gain and weight status was assessed through a logistic regression analysis, adjusting for gender, age group, household composition, employment and quality of life. Odds Ratio (OR), the 95% confidence interval (CI) and the *P*-values are reported in the text.

Logistic regression models were used to determine the associations between self-reported weight gain as the dependent variable and health behaviour change indicators as independent variables (health behaviour change indicators included, whether associated with the outcome or not), adjusted for gender, age group, household composition, employment status, quality of life and the health behaviour indicators. Crude and adjusted ORs with 95% confidence intervals (CIs) and *P*-values were presented in a table; the adjusted ORs are reported in the text. Since health behaviours may differ by gender or age group (3 groups), additional stratified analyses by those covariates separately were also conducted. All the analyses were performed with SAS® 9.4 [28] using the PROC SURVEY-procedures, taking the post-stratification weights into account.

## Results

### Characteristics of the study population

Table 2 presents the distribution of the characteristics of the study population. Overall, 28.6% of the persons aged 18 years and older in Belgium reported to have gained weight in the first 6 weeks of the confinement, 56.9% reported their weight remained stable and 14.5% reported to have lost weight. The overall prevalence of those who have changed at least one health behaviour, positively or negatively, was 82,8%; in case of negatively this was 58,5%. The most frequently reported behaviour changes in these 6 weeks (Table 2) were an increased consumption of sweet or salty snacks (33.2%) and a decrease in physical activity (28.8%).

**Table 2:** Distribution of the study population (*N* = 28,029) by sociodemographic covariates and change in self-reported health behaviours in 6 weeks during confinement, second COVID-19 Health Survey, Belgium 2020

Background variables and indicators	Crude prevalence (%)	Weighted^**a**^ prevalence (%)
SOCIODEMOGRAPHIC AND HEALTH COVARIATES		
Gender		
Men	32.5	50.9
Women	67.5	49.1
Age group		
18–24 years	2.7	11.7
25–34 years	15.0	15.7
35–44 years	23.5	17.0
45–54 years	24.1	18.4
55–64 years	22.0	17.3
65+ years	12.7	19.9
Household composition		
Living alone	15.6	16.7
Couple, without child (ren)	29.5	31.9
Couple, with child (ren)	40.1	30.5
Living alone with child (ren)	6.9	4.7
Living with parents, family, …	6.8	15.2
Other	1.1	1.0
Employment		
No paid job	23.8	37.7
Paid job, normal environment	21.7	21.0
Paid job, but via telework	40.1	27.2
Paid job, but temporarily unemployed	7.8	9.3
Paid job, other situation	6.6	4.8
Reporting no health problem (EQ-5D - quality of life)		
Yes	27.8	27.1
No	72.2	72.9
Region		
Flemish	53.2	60.7
Brussels	10.5	9.4
Walloon	36.3	29.9
SELF-REPORTED WEIGHT STATUS AND CHANGE IN HEALTH BEHAVIOUR IN 6 WEEKS INDICATORS		
Weight status		
Underweight	2.1	2.4
Normal weight	47.8	44.8
Overweight	32.7	34.2
Obesity	17.4	18.6
Change in body weight		
Lost weight	14.1	14.5
Weight remained stable	56.1	56.9
Gained weight	29.8	28.6
Change in the consumption of sugared-sweetened beverages		
Increased	8.7	9.2
Unchanged	84.2	82.1
Decreased	7.1	8.7
Change in the consumption of sweet or salty snacks		
Increased	36.6	33.2
Unchanged	56.8	59.4
Decreased	6.6	7.4
Change in the consumption of food prepared out-of-home		
Increased	6.6	7.3
Unchanged	51.8	53.0
Decreased	41.6	39.7
Change in physical activity		
Increased	24.6	23.7
Unchanged	45.1	47.5
Decreased	30.3	28.8
Change in alcohol consumption		
Increased	21.2	17.4
Unchanged	65.0	64.9
Decreased	13.8	17.7

^a^Weighted for age, gender, education and province

### Weight change according to weight status

**Fig. 1:**
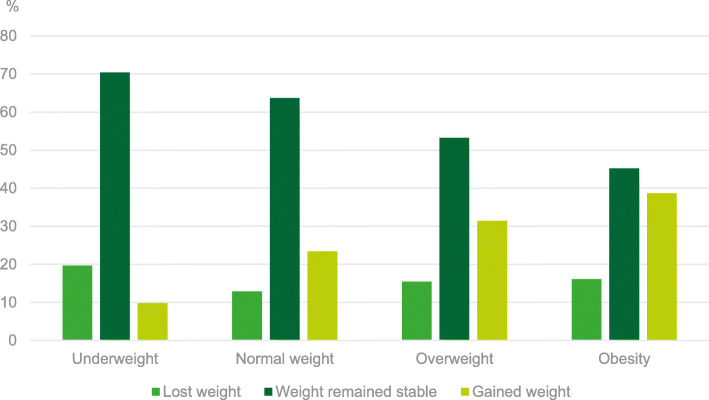
Distribution (%) of the self-reported weight change in 6 weeks during confinement according to the self-reported weight status, second COVID-19 Health Survey, Belgium 2020

Figure 1 shows that the proportion of persons who reported some weight gain in the first 6 weeks of the confinement increased with the increasing BMI categories: weight gain was reported by 9.9% of the persons who are underweight, 23.4% of the persons with a normal weight, 31.4% of the persons with overweight and 38.7% of the persons with obesity.

Compared to persons in the normal (healthy) weight range, the odds of gaining weight was higher for overweight persons (OR = 1.72 (1.56–1.91), *P*-value< 0.0001) and those with obesity (OR = 2.23 (1.98–2.52), *P*-value< 0.0001), and lower for underweight persons (OR = 0.30 (0.19–0.46), P-value< 0.0001).

### Association between health behaviour and weight gain

Table 3 shows that persons with an increased consumption of sugar-sweetened beverages in the first 6 weeks of the confinement had higher odds of weight gain (OR_adj_ = 1.39 (1.15–1.68), *P*-value< 0.001). However, persons who decreased their consumption of sugar-sweetened beverages also had higher adjusted odds of weight gain (OR_adj_ = 1.29 (1.04–1.60), *P*-value = 0.018). The highest odds of weight gain was observed for persons with an increased consumption of sweet or salty snacks in these 6 weeks (OR_adj_ = 3.65 (3.27–4.07), *P*-value< 0.0001). Furthermore, persons who were less physically active in the 6 weeks of confinement were almost twice as likely to gain weight (OR_adj_ = 1.91 (1.71–2.13), *P*-value< 0.0001). Persons who were more active in these 6 weeks had lower odds of gaining weight in the adjusted model only (OR_adj_ = 0.84 (0.74–0.96), *P*-value = 0.013). Finally, an increased consumption of alcohol in these 6 weeks was positively associated with weight gain (OR_adj_ = 1.86 (1.66–2.08), *P*-value< 0.0001). Persons who decreased their consumption had a lower adjusted odds of weight gain (OR_adj_ = 0.85 (0.72–0.99), *P*-value = 0.043).

**Table 3:** Association between self-reported weight gain in 6 weeks during confinement and change in self-reported health behaviours (*N* = 28,029) by means of crude and adjusted^a^ OR (95% CI) and *P*-value, second COVID-19 Health Survey, Belgium 2020

Self-reported weight gain by change in self-reported health behaviour	Crude			Adjusted^a^		
	OR	95% CI	*P*-value	OR	95% CI	*P*-value
Change in the consumption of sugared-sweetened beverages (Reference = Unchanged)						
Increased	2.62	2.24–3.06	<.0001	1.39	1.15–1.68	0.0006
Decreased	1.09	0.90–1.30	0.375	1.29	1.04–1.60	0.022
Change in the consumption of sweet or salty snacks (Reference = Unchanged)						
Increased	4.58	4.16–5.04	<.0001	3.65	3.27–4.07	<.0001
Decreased	0.95	0.76–1.19	0.660	0.86	0.66–1.12	0.262
Change in the consumption of food prepared out-of-home (Reference = Unchanged)						
Increased	1.94	1.63–2.30	<.0001	1.20	1.00–1.45	0.051
Decreased	1.17	1.07–1.29	0.001	0.95	0.86–1.06	0.390
Change in physical activity (Reference = Unchanged)						
Increased	0.99	0.88–1.12	0.894	0.84	0.74–0.96	0.013
Decreased	2.32	2.10–2.57	<.0001	1.91	1.71–2.13	<.0001
Change in alcohol consumption (Reference = Unchanged)						
Increased	2.64	2.39–2.92	<.0001	1.86	1.66–2.08	<.0001
Decreased	0.95	0.82–1.10	0.517	0.85	0.72–0.99	0.043

^a^Adjusted for age, gender, household composition, employment and the health behaviour indicators

### Association between weight gain and health behaviour stratified by gender

Overall, the associations between changes in health behaviours and weight gain were similar for men and women, although two differences were observed (Table 4): women who increased their consumption of food prepared out-of-home had higher adjusted odds of weight gain in the first 6 weeks of confinement than men (OR_adj_ = 1.28 (1.02–1.61), *P*-value = 0.036, and OR_adj_ = 1.14 (0.85–1.51), *P*-value = 0.381, respectively) and men who were more physically active in these 6 weeks had lower adjusted odds of weight gain than women (OR_adj_ = 0.77 (0.61–0.98), *P*-value = 0.033, and OR_adj_ = 0.91 (0.79–1.05), *P*-value = 0.195, respectively).

**Table 4:** Association between self-reported weight gain in 6 weeks during confinement and change in self-reported health behaviours by means of adjusted^a^ OR (95% CI) and *P*-value, stratified by gender, second COVID-19 Health Survey, Belgium 2020

Self-reported weight gain by change in self-reported health behaviour	Men (***N*** = 9296)			Women (***N*** = 19,369)		
	OR	95% CI	*P*-value	OR	95% CI	*P*-value
Change in the consumption of sugared-sweetened beverages (Reference = Unchanged)						
Increased	1.44	1.03–2.01	0.034	1.37	1.14–1.64	0.0007
Decreased	1.36	0.98–1.91	0.068	1.18	0.91–1.52	0.219
Change in the consumption of sweet or salty snacks (Reference = Unchanged)						
Increased	3.22	2.66–3.91	<.0001	4.05	3.60–4.56	<.0001
Decreased	0.75	0.49–1.16	0.196	0.98	0.71–1.34	0.876
Change in the consumption of food prepared out-of-home (Reference = Unchanged)						
Increased	1.14	0.85–1.51	0.381	1.28	1.02–1.61	0.036
Decreased	1.00	0.83–1.21	0.964	0.92	0.82–1.04	0.184
Change in physical activity (Reference = Unchanged)						
Increased	0.77	0.61–0.98	0.033	0.91	0.79–1.05	0.195
Decreased	2.15	1.80–2.57	<.0001	1.69	1.49–1.93	<.0001
Change in alcohol consumption (Reference = Unchanged)						
Increased	1.86	1.54–2.45	<.0001	1.84	1.62–2.10	<.0001
Decreased	0.85	0.67–1.08	0.183	0.83	0.68–1.02	0.070

^a^Adjusted for age, household composition, employment and the health behaviour indicators

### Association between health behaviour and weight gain stratified by age group

Some of the associations between health behaviours and weight gain appear to differ in intensity across age groups (Table 5). In particular, an increased alcohol consumption was more strongly associated with weight gain in older adults (OR_adj_ = 2.52 (2.03–3.12), *P*-value< 0.0001). The association between increased snacking and weight gain also appears to strengthen with age (OR_adj_ = 3.92 (3.29–4.68), *P*-value< 0.0001). An increased consumption of food prepared out-of-home appears only to be influencing weight gain in the middle age group (OR_adj_ = 1.27 (1.00–1.60), *P*-value = 0.048), while a decrease in sugared-sweetened beverages appears to be mostly associated with weight gain in older adults (OR_adj_ = 1.55 (1.09–2.20), *P*-value = 0.016).

**Table 5:** Association between self-reported weight gain in 6 weeks during confinement and change in self-reported health behaviours by means of adjusted^a^ OR (95% CI) and *P*-value, stratified by age group, second COVID-19 Health Survey, Belgium 2020

Self-reported weight gain by change in self-reported health behaviour	Young adults 18–34 years (***N*** = 5105)			Middle aged adults 35–54 years (***N*** = 13,617)			Older adults 55+ years (***N*** = 9943)		
	OR	95% CI	*P*-value	OR	95% CI	*P*-value	OR	95% CI	*P*-value
Change in the consumption of sugared-sweetened beverages (Reference = Unchanged)									
Increased	1.34	0.95–1.90	0.095	1.48	1.23–1.78	<.0001	1.33	0.90–1.97	0.148
Decreased	1.18	0.79–1.77	0.408	1.21	0.93–1.58	0.159	1.55	1.09–2.20	0.016
Change in the consumption of sweet or salty snacks (Reference = Unchanged)									
Increased	3.22	2.48–4.18	<.0001	3.73	3.29–4.23	<.0001	3.92	3.29–4.68	<.0001
Decreased	0.99	0.57–1.72	0.977	0.64	0.47–0.88	0.006	0.93	0.63–1.37	0.697
Change in the consumption of food prepared out-of-home (Reference = Unchanged)									
Increased	1.15	0.78–1.70	0.477	1.27	1.00–1.60	0.048	1.09	0.79–1.50	0.615
Decreased	0.94	0.73–1.21	0.624	0.88	0.77–0.99	0.040	1.07	0.89–1.30	0.471
Change in physical activity (Reference = Unchanged)									
Increased	0.73	0.54–0.98	0.039	0.81	0.70–0.94	0.007	0.98	0.79–1.22	0.878
Decreased	1.54	1.19–2.01	0.001	2.10	1.83–2.40	<.0001	2.00	1.67–2.39	<.0001
Change in alcohol consumption (Reference = Unchanged)									
Increased	1.40	1.08–1.80	0.010	1.83	1.60–2.09	<.0001	2.52	2.03–3.12	<.0001
Decreased	0.90	0.65–1.23	0.504	0.84	0.69–1.03	0.090	0.73	0.57–0.94	0.014

^a^Adjusted for gender, household composition, employment and the health behaviour indicators

## Discussion

This study assessed the association between weight gain and changes in health behaviours, such as nutritional habits, physical activity and alcohol consumption, during the 6-weeks confinement period. More than a quarter (28.6%) of the adults reported weight gain over this period in Belgium. Persons who already suffered from overweight or obesity reported weight gain more frequently. Weight gain during confinement has also been reported in other studies: 22% of adults in the US sampled by Facebook reported gaining weight during self-quarantine due to COVID-19 [29] and 49% of the Italians (survey organised between the 5th and 24th of April 2020, after 7 weeks of confinement) [15].

An increased consumption of sweet or salty snacks and being less physically active during this period both appear to be important health behaviour changes associated with weight gain during the confinement period. These behaviours were also found to be major risk factors in other studies [2, 3, 29, 30]. Eating unhealthy food and being physically inactive tend to cluster [31].

The proportion of persons who indicated having increased their consumption of food prepared out-of-home in the first 6 weeks of confinement is low. Nevertheless, an increased consumption of alcohol was found to be a risk factor for weight gain in 6 weeks during confinement, especially in the older age groups. The closure of bars and restaurants undoubtedly had an impact on the consumption of alcohol, especially for social drinkers and youngsters who could not go out anymore whereby their lower consumption. However, confinement also results that people will drink more (often) alcohol at home.

Confinement and other COVID-19 related restrictions substantially altered the social, physical and economic environments in which people lived, which resulting in a modification of health behaviours for many. While some people had the social, economic and educational resources to make healthier (food) choices, other people adopted less healthy (food) behaviours and gained weight as a result [16, 32]. In the subsequent epidemic wave, it is necessary that policy makers pay a greater attention to these unintended consequences, so that the prevalence of overweight and obesity does not continue to increase. According to this second COVID-19 health survey during the confinement, 18.0% of the Belgium adults were classified as obese, a prevalence that was significantly higher than that of the national Health Interview Survey in 2018 (15.9%) [18]. It will be important to consider our eating habits, especially with regard to the consumption of sweet and salty snacks. A balanced diet, rich in nutrients and antioxidants, not only helps controlling our body weight [7–9], it also helps to have a strong immune system [15, 33, 34]. It is crucial, especially during confinement, to keep good dietary habits including fresh fruits, vegetables, whole grains, plant and animal protein and healthy fats. In addition, hydration is important and water is the healthiest and cheapest way to do this [34]. A Belgian study has shown that food insecurity during confinement was associated with adverse changes in dietary habits and that support of the government is needed to tackle it [32]. Beyond the direct effect of unhealthy eating and increased obesity during the confinement measures, the COVID-19 pandemic further amplified the burden of obesity by more severely affecting people with overweight or obesity. This highlights the need for more ambitious policies to address the multiple determinants of obesity and unhealthy eating in Belgium. Potential policy actions could be labelling to help people making healthy food choices, legislation to end the promotion of foods high in fat, sugar or salt (HFSS) and banning the advertising of HFSS products on TV and online.

Besides a healthy diet, staying active during confinement is also an important health behaviour, not only for controlling the weight status, but also for the well-being and the quality of life [35]. In Belgium, even with the confinement measures, the population still had the opportunity to go outside, but in their local environment. Additionally, the combination of good weather conditions during this period and more free time due to a change in the work situation for some people made it easier to be active. This was also observed in this survey since 47.5% of the population has indicated that their physical activity habits remained the same and even 23.8% was more physically active in this period. Nevertheless, 28.8% of the adults were less physically active in this period, which could be attributed to confinement measures such as closure of indoor sport facilities, more sedentary time spent in front of a computer, or the burden of home schooling because of school closure. To avoid similar reductions in physical activity as new restrictions measures are imposed to curb subsequent COVID-19 waves, outdoor physical activity should be actively promoted.

This study has several strengths. Firstly, the online tool made it possible to react rapidly to the crisis. The first COVID-19 health survey was launched only 3 weeks after the confinement. A web survey not only has financial advantages, but also logistical ones (automatic data entry, user-friendly by checks and automatic branching logic) whereby high quality data were instantly available [36, 37]. Moreover, the survey could be answered on several devices like a mobile phone, a tablet and computer that makes it very accessible. Another strength is that a large sample of the population aged 18 years and older was collected on a convenience sample. The weakness of this fast method of sampling is that it concerns a more biased process since there is no randomisation [26]. Consequently, the composition of our sample differed from the composition of the general Belgian population aged 18 years and older. An overrepresentation of women and higher educated people, as well as an underrepresentation of elderly was also established in the French NutriNet-Santé cohort study who also applied weights in the analyses to improve the representativeness of the population [2]. Besides elderly and low educated people are also the groups that are less motivated to participate in other web surveys [38]. In addition to this selection bias, there is also a potential healthy volunteer bias since the survey was advertised, among others, on the Sciensano website and the respondents who visit this website are probably more interested in this research area. Another weakness of this study is that self-reported data may be related to misreporting [15]. It is well known that the BMI based on self-reported measures is often underestimated [6]. Lastly, the questions related to ‘change in’ (behaviours or status) were specifically developed for this survey without pre-testing (due to the emergency of the COVID-19 situation), which could have an influence on their reliability.

## Conclusion

The results from this study may help the government to determine specific strategies to prevent a further increase in the prevalence of overweight and obesity if a similar crisis occur or new confinement measures are introduced due to COVID-19 in the future. This is important since overweight and obese people not only have an increased risk of morbidity (cardiovascular diseases, diabetes type 2 and some cancers) and premature death, but recent studies have shown that obesity increases the risk of severe COVID-19 which may result in an increased pressure on the health care system.

## Data Availability

Access to the data of the second Belgian COVID-19 health survey can be requested by sending an e-mail to HIS@sciensano.be.

## Abbreviations

BMI: Body mass index
OR: Odds Ratio
CI: Confidence interval
HFSS: foods high in fat, sugar or salt

## Footnotes

1 Unemployment, invalidity, studies, retirement, household work and other situation
